# Factors shaping pelagic-benthic coupling in the process of settlement in an Arctic fjord

**DOI:** 10.1038/s41598-024-74062-8

**Published:** 2024-10-10

**Authors:** Marta Ronowicz, Piotr Balazy, Maciej Chełchowski, Piotr Kuklinski, Weronika Patuła, Anna Sowa, Janne E. Søreide, Agata Weydmann-Zwolicka

**Affiliations:** 1 Marine Ecology Department, Institute of Oceanology, Polish Academy of Sciences, Sopot, Poland; 2 Department of Arctic Biology, The University Centre in Svalbard, Longyearbyen, Norway; 3 Laboratory of Plankton Biology, Department of Marine Biology and Biotechnology, University of Gdansk, Gdynia, Poland

**Keywords:** Pelago-benthic coupling, Epibenthos, Meroplankton, Larvae, Svalbard, Spitsbergen, Isfjorden, Marine biology, Ecology

## Abstract

Benthic organisms typically possess a planktonic propagule stage in the form of larvae or spores, which enables them to spread over large distances before settlement, and promotes tight pelago-benthic coupling. However, factors driving dispersal and epibenthos recruitment in shallow hard-bottom Arctic communities are poorly known. We therefore conducted a year-round in situ colonization experiment in Isfjorden (Svalbard), and found out that variation in early-stage epibenthic assemblages was explained by the combination of: abiotic (45.9%) and biotic variables (23.9%), and their interactions (30.2%). The upward-facing experimental plates were dominated by coralline algae, and this is the first study showing that at high latitudes coralline algae *Lithothamnion* sp. settle in high numbers on available substrates during the polar night in winter. The downward-facing plates, which had much less exposure to light, contained more diverse organisms, with a predominance of polychaetas and bryozoans. However, in summer, the barnacle *Semibalanus balanoides* outcompeted all the other recruits, as a result of massive occurrence of meroplanktonic Cirripedia larvae, triggered by the phytoplankton bloom. In conclusion, the rate and success of epibenthic settlements were dependent mostly on light availability and temperature, suggesting that larval settlement will be impacted by global warming with some taxa benefitting, while others losing.

## Introduction

Most benthic invertebrates, for a restricted time in their life cycles, possess a pelagic larval stage, which seasonally supplies the zooplankton community in high numbers, comprising the temporary fraction called meroplankton. Larval release by benthic animals, or spores by benthic algae, into the water column and their further fate and recruitment on the seafloor are examples of bidirectional pelago-benthic coupling^1^, the process connecting pelagic and benthic ocean realms by the flow of matter. The possession of larval stages enables the spread of otherwise stationary benthic species and allows the colonization of new territories, which again lowers intraspecific food competition^2^, reduces inbreeding and facilitates the preservation of genetic diversity^3^. This interconnection between benthos and pelagos largely depends on physical and biological environmental variables, such as community structure and reproductive potential, seasonality, bottom topography, local hydrology (ocean tides and currents), transport mechanisms, depth and ice formation in polar regions^1^, and primary production, as spring blooms are often a triggering mechanism for releasing larvae^4^.

While the benthic zone is considered a stable system with low variability in temperature, salinity, light and water currents, the pelagic zone is seasonally highly variable, especially in regard to sea ice, light conditions and primary production^5^. The larval phase thrives in the 3-dimensional environment of the water column, and allows the dispersal of benthic organisms across wide distances. Depending on whether the larva has energy storages (lecithotrophic) or is dependent on feeding (planktotrophic), the propagule duration can vary from a few minutes to months^6^. Larvae that stay in the water column for a shorter period colonize areas close to maternal organisms, in contrast to larvae that stay in the water column for a longer period; the latter type of larvae will be able to colonize broad areas, often kilometres away from their parents^7^. Dispersal, however, is not a simple function of larval duration, as the behaviour of larvae can strongly influence dispersal distance^6^; for instance, larvae that remain close to the bottom, where there is a slower water current disperse shorter distances^6^. Therefore, on the one hand, the distribution of benthic adults determines the supply of larvae, and on the other hand, the larval pool influences adult occurrence and thus the structure of benthic communities^8^.

Due to global warming, the Arctic is under the influence of increasingly warm Atlantic water inflow^9^. The continuous transport of organisms of boreal origin through the North Atlantic Currents and further through the West Spitsbergen Current, combined with higher sea temperatures and less sea ice, has led to the expansion of sub-Arctic and boreal populations in the Arctic^10,11^. The West Spitsbergen region, which is the study area for this investigation, is also highly exposed to advection processes^12–14^, where spatial decoupling between larval production and local settlement may occur^15^. For many invertebrates, there is a tight relationship between water temperature and spawning time, including the optimal temperature range for development^16^. The larval and juvenile stages are particularly vulnerable to environmental stressors, and with higher sea temperatures, these stages may be weak links for the survival of some species^17^. The temperature-size rule^18^ states that increasing temperature accelerates developmental rates to be faster than growth rates, resulting in smaller body sizes^19^. Therefore, it is critical to study the connection between meroplankton and benthos, as well as the factors shaping the pivotal process of recruitment, to predict the possible impacts of global warming on benthic larval development and settlement. Such an approach has rarely been implemented, especially when accessing the Arctic region is logistically difficult.

In recent years, several studies have focused on the seasonal dynamics of meroplankton^20–23^, and early benthic community development in the Arctic coastal waters^24–26^; however, these components have seldom been examined simultaneously, which restricts us from making comparisons and drawing direct correlations between the pelagic and benthic zones in this region. The exceptions include the seasonal pioneer study by Kuklinski et al.^27^, which aimed to connect the pelagic larval stage with invertebrate settlement in the high Arctic Isfjorden (Spitsbergen), and other interesting investigations on connectivity between meroplankonic larvae and benthic adults carried out in the Pacific Arctic by Ershova et al.^15^ and Meyer-Kaiser et al.^28^. Ershova et al.^15^ revealed a mismatch between larval and benthic communities during the summer months, explained by advection being the main driver of larval distribution. Meyer-Kaiser et al.^28^ revealed the differences in the species composition of meroplankton and fouling organisms sampled opportunistically between the Atlantic Water and Arctic Water in the Fram Strait, highlighting the importance of the West Spitsbergen Current for larval transport. However, seasonal patterns were not explored in these two studies.

Therefore, the aim of the present study, based on year-round seasonal meroplankton sampling and environmental measurements, coupled with a colonization experiment conducted in the shallow hard bottom region of a high-Arctic fjord, Isfjorden, was to contribute to a better understanding of complex interactions between the environment and biotic components of ecosystems, particularly the recruitment of sessile benthic biota. The main goal was to identify the driving factors influencing the recruitment of epibenthic organisms in the shallow hard-bottom region of the Arctic fjord, with a special emphasis on the connectivity between pelagic and benthic life history stages.

## Materials and methods

### Ethical statement

Sample collection was undertaken on non-endangered species which had no significant impact on their populations at the site. The study complies with the Guidelines for researchers in Svalbard required by the Governor of Svalbard.

### Study area

The study was conducted in Isfjorden, which is a widely open (without a shallow sill at the entrance) fjord system, the largest on the western coast of Spitsbergen (Svalbard Archipelago). Isfjorden has a broad range of hydrologic conditions and diverse underwater landscapes, including a soft bottom with high sedimentation in the innermost, glacier-impacted bays, to the rocky bottom and more clear-water outer areas. Due to the influence of waters of both Arctic and Atlantic origin, as well as freshwater runoff from melting glaciers and large rivers, the salinity in shallow areas varies from 28 to 34^23,29^. The sea water temperature ranges from − 1.4 to 7 °C^23,30^. Sea ice covers the inner bays of the northern and eastern fjord regions from November to July^31^. The average amplitude of tides is 130 cm^32^. The polar night lasts 112 days in the area (data from https://sunsetsunrisetime.com).

**Fig. 1:**
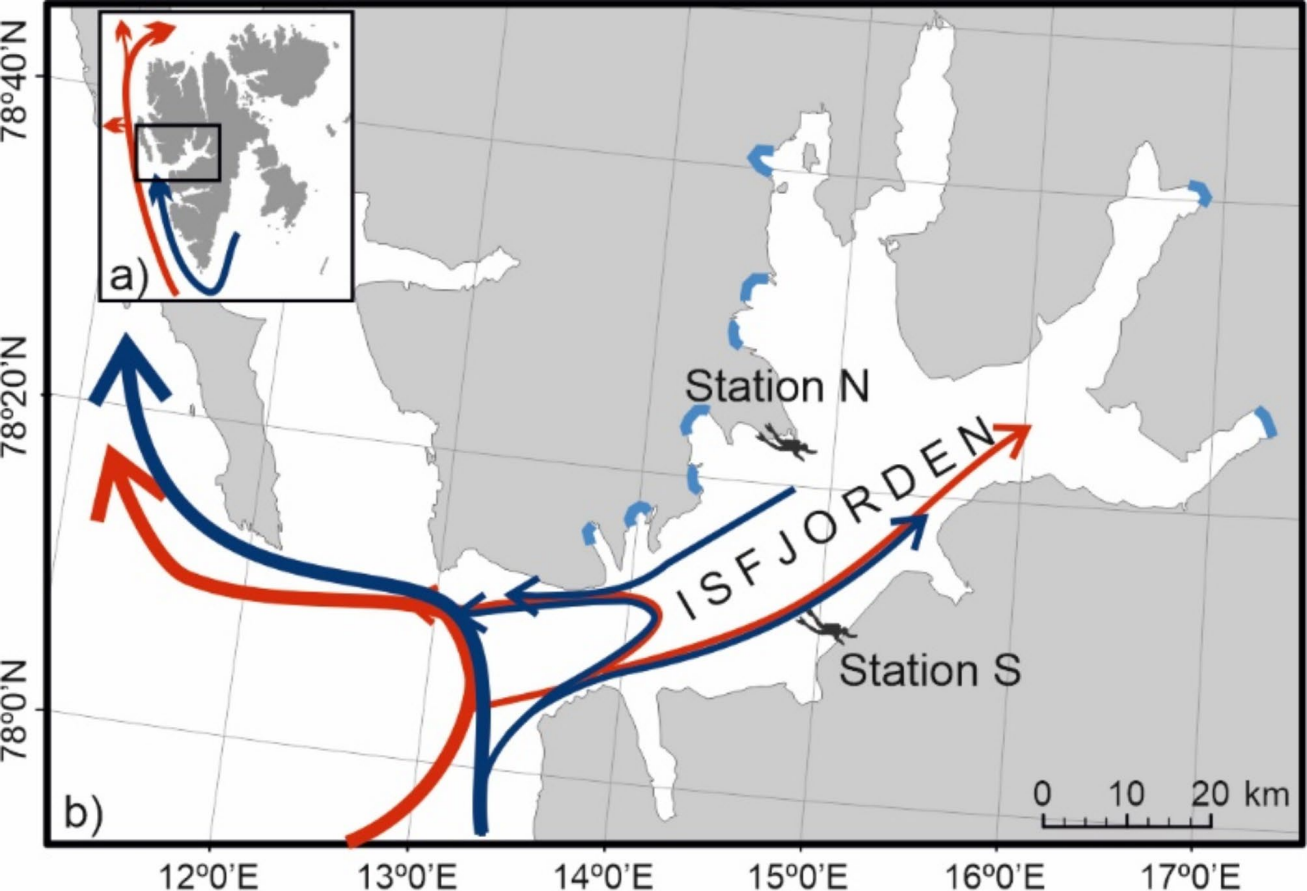
Study area: (**a**) Svalbard Archipelago with major ocean currents shown: the Arctic Water carried by the South Cape Current (dark blue arrow), and the Atlantic Water of the West Spitsbergen Current (red arrow); (**b**) the location of the two sampling stations (southern [S], and northern [N]) in Isfjorden, where the inflow of the Transformed Atlantic Water normally occurs along the southern side of the fjord (red arrows), which is compensated for by the Local Water (dark blue arrows) being pushed out on the northern side. Glacier fronts are shown as blue lines along the coastline. The circulation pattern was modified based on Nilsen et al.^29^ and Weydmann-Zwolicka et al.^23^. (The map was created with the use of CorelDRAW 2019).

The two study stations, northern-N (78° 22,585′ N; 14° 46,929′ E) and southern-S (78° 11,299′ N; 15° 08,685′ E; Fig. 1), differ in their environmental conditions, mostly due to the anticlockwise circular movement of sea water, which flows from the shelf along the southern coast and exits on the northern side^33^, as well as the distance from tidewater glaciers, bathymetric conditions and seabed characteristics. Station N is more Arctic in nature with slightly lower sea water temperatures, more sea ice present and more fresh water from melting glaciers^23^. The seabed is relatively flat and deepens slowly towards the fjord centre. It consists of a mixture of cobbles and boulders lying on mud and is an attachment point for macroalgae. The bottom at Station S is a hard bedrock with steep rocky shelves down to 15 m depth, after which the bedrock flattens. The substrate was a similar mixture of cobbles, boulders and sediment patches. On hard bedrock, a dense kelp forest consists mostly of three species, *Saccharina latissima*, *Alaria esculenta* and *Laminaria* spp., while polychaetes, barnacles, bryozoans, cnidarians (in large numbers, *Urticina crassicornis*, *Hormathia nodosa*, *Cribrinopsis similis*,* C. olegi*), ascidians (*Halocynthia* sp.) and sponges are found underneath. Mobile benthic fauna include hermit crabs (*Pagurus pubescens*), spider crabs (*Hyas* spp.), whelks (*Buccinum* spp.), shrimps (*Lebbeus polaris*, *Eualus gaimardii*, *Sclerocrangon boreas*), sea stars (*Crossaster papposus*, *Henricia* sp.), and sea urchins (*Strongylocentrotus droebachiensis*). Bottom-dwelling fish, such as the sculpin *Myxocephalus scorpius*, the wolfish *Anarhichas lupus*, and more and more frequently young individuals of the Atlantic cod *Gadus morhua*, which use these areas as nursery grounds, are also found at this station. The vast majority of these species also occur at Station N but not at such large numbers or densities (personal observations).

### Sampling

Field experiments and sampling were performed by the IO PAN Scientific Diving Team every 3 months from July 2016 to July 2017 at both stations and two depths (Table 1), with the first expedition organized to set up the experimental panel constructions. The experimental panels were composed of upward- and downward-facing triplicate settlement plates (15 cm × 15 cm) attached to a metal frame (for a detailed description of the construction, see Kuklinski et al.^34^) and submerged at two depths (6 and 12 m) at both stations for three months. Temperature and light intensity loggers (HOBO Pendant Temperature/Light 64 K) were used for every experimental construction, and measurements were taken continuously throughout the year at 30-min intervals. At each of the four sampling campaigns, the experimental plates were recovered, preserved in 96% ethanol, and replaced with new plates.

**Table 1:** Dates of sampling at each station and depth in Isfjorden in 2016–2017.

Season	Station			
	S		N	
	Depth			
	6 m	12 m	6 m	12 m
Summer 1	28.07.16	27.07.16	1.08.16	1.08.16
Autumn	11.10.16	12.10.16	16.10.16	13.10.16
Winter	23.01.17	14.01.17	n.d.	17.01.17
Spring	28.04.17	28.04.17	1.05.17	26.04.17
Summer 2	18.07.17	18.07.17	20.07.17	20.07.17

n.d.—no data; underwater experimental construction not found due to harsh weather conditions.

Every 3 months (Table 1) the following sampling strategy was performed, as described in Weydmann-Zwolicka et al.^23^. Water samples for chlorophyll *a* (Chl *a*) and phytoplankton (planktonic protists) community analyses were collected in 1 dm^3^ containers from the surface (0 m) and at the bottom at each study station. At least 100 ml of water was filtered through Whatman GF/F glass fibre filters and frozen at – 80 °C for later Chl *a* determination. Phytoplankton samples for the taxonomic analysis were preserved in buffered 2% formaldehyde solution. To representatively collect zooplankton, including potential recruits belonging to meroplankton, and demersal fauna two sampling methods were applied. First, a handheld net with an opening of 0.25 m^2^ and a mesh size of 100 μm (modified Stanwell-Smith et al.^35^) was towed horizontally approximately one meter above the bottom. The net was towed each time for 100 m (back and forth four times for 25 m). Second, an underwater suction pump with a mesh size of 100 μm was maneuvered for 10 min over an area of approximately 2 m^2^^23^. The zooplankton samples were preserved in 96% ethanol immediately after collection.

### Sample analysis

The central 100 cm^2^ areas (10 cm × 10 cm) of the experimental plates were analysed under a Leica M205C stereomicroscope to eliminate the ‘edge effect’ and ensure that the results were comparable with those of other studies. The epibenthic assemblages were identified to the lowest possible taxonomic level. Due to the short submergence period (3 months), many of the organisms were still in their juvenile developmental stages, hindering identification at the species level. For the purpose of identifying Spirorbinae (Polychaeta), morphology-based categories were used. Organisms coiling to the left were assigned to *Circeis* sp., those coiling to the right to *Spirorbis* sp./*Bushiella* sp./*Pilleolaria* sp., and those that did not yet form a coil were placed in the Spirorbinae juvenile category. During this study, proper identification of serpulids could not be performed due to their early stage of development. Colonies of bryozoans were considered singular organisms.

The concentrations of Chl *a* were determined within three months using methanol as the extraction solvent and were measured with a Turner Design AU-fluorometer (calibrated with pure Chl *a*; Sigma S6144). Phytoplankton taxonomic samples were analysed according to the procedure described by Utermӧhl^36^. After 24 h of settlement in the Utermӧhl counting chamber, the cells were identified and counted under an inverted Nikon TE300 microscope. Chl *a* was extracted in 96% ethanol in the dark for 24 h and determined spectrophotometrically (Beckman DU 68). The concentrations were calculated based on the equation of Jeffrey and Humphrey^37^. Zooplankton samples were analysed under a Nikon SMZ 1000 binocular microscope for taxonomic composition following the slightly modified procedure of Postel et al.^38^, as described by Weydmann-Zwolicka et al.^23^.

### Data analysis

For statistical analyses, the epibenthic taxa abundance data from each experimental set (consisting of three plates), were averaged for the plates facing the water column (up, U) and the ones facing the sea bottom (down, D). This approach yielded 30 samples in total that were treated as compositional samples in the following analyses, which were performed on log-transformed [x′ = log (x + 1)] abundance data of different epibenthic taxa noted on the plates. The significance level of all the following statistical tests was set at *p* ≤ 0.05. To reveal the main processes influencing the recruitment of epibenthic animals and algae in shallow Arctic waters, the relationships between the abundance of epibenthic taxa and environmental (abiotic and biotic) variables were analysed using two different approaches.

First, the epibenthic taxonomic composition was investigated using linkage tree analysis (LINKTREE) with a series of similarity profile (SIMPROF) tests applied in PRIMER 7^39^. This method involves the divisive clustering of samples constrained by inequalities in one or more biotic variables; in this case, the epibenthic taxonomic composition was also included. Thus, the samples with characteristic epibenthic taxa were divided into smaller groups, where each division had an ‘explanation’ regarding the threshold on their abundance. The following set of conditions provided the best linkage tree illustration: minimum group size = 3, minimum split size = 3, and minimum split *R* = 0.5.

Second, we applied a series of six redundancy analyses (RDAs) in Canoco 5^40^ to study the relationships between the epibenthic taxonomic composition and environmental variables, both abiotic and biotic, separately for each set of explanatory variables, including (1) environmental/abiotic conditions, (2) potential recruits, (3) zooplankton/demersal taxa, (4) phytoplankton at the start of plate exposition, (5) potential recruits and (6) zooplankton/demersal taxa at the end of plate exposition. The tested abiotic predictors included temperature and light intensity, which were measured continuously by HOBO data loggers and averaged for the period of experimental panel exposure; settlement plate position (upward, U or downward, D); sampling depth (6–12 m); and station (N or S). The biotic factors included binarized data on the presence/absence of potential recruits (here understood as meroplankton) and zooplankton, including demersal taxa, which were sampled by SCUBA diver-operated devices, horizontal towed net and underwater suction pump at the start and end of panel exposure, according to the protocols described in Weydmann-Zwolicka et al.^23^. We assumed that taxa listed as meroplankton may act as potential recruits for the epibenthic community, while planktonic (including phytoplankton and holoplankton) and demersal organisms may be potential food sources. For the beginning of panel exposure, phytoplankton data were also included. This approach resulted in the testing of six sets of environmental explanatory variables, which were ranked according to their quantitative importance by forward selection and were the basis of the subsequent variation partitioning between abiotic and biotic factors.

In the end, we applied variation partitioning to the RDA and tested for conditional effects to disentangle the contributions of two groups of environmental variables: abiotic (temperature, light intensity, settlement plate position, sampling depth and station) and biotic (selected taxa of potential recruits, zooplankton, and phytoplankton collected during panel deployment; as well as potential recruits and zooplankton collected at the end of exposure), which significantly affected the composition of epibenthic taxa according to the previous RDAs performed.

## Results

**Fig. 2:**
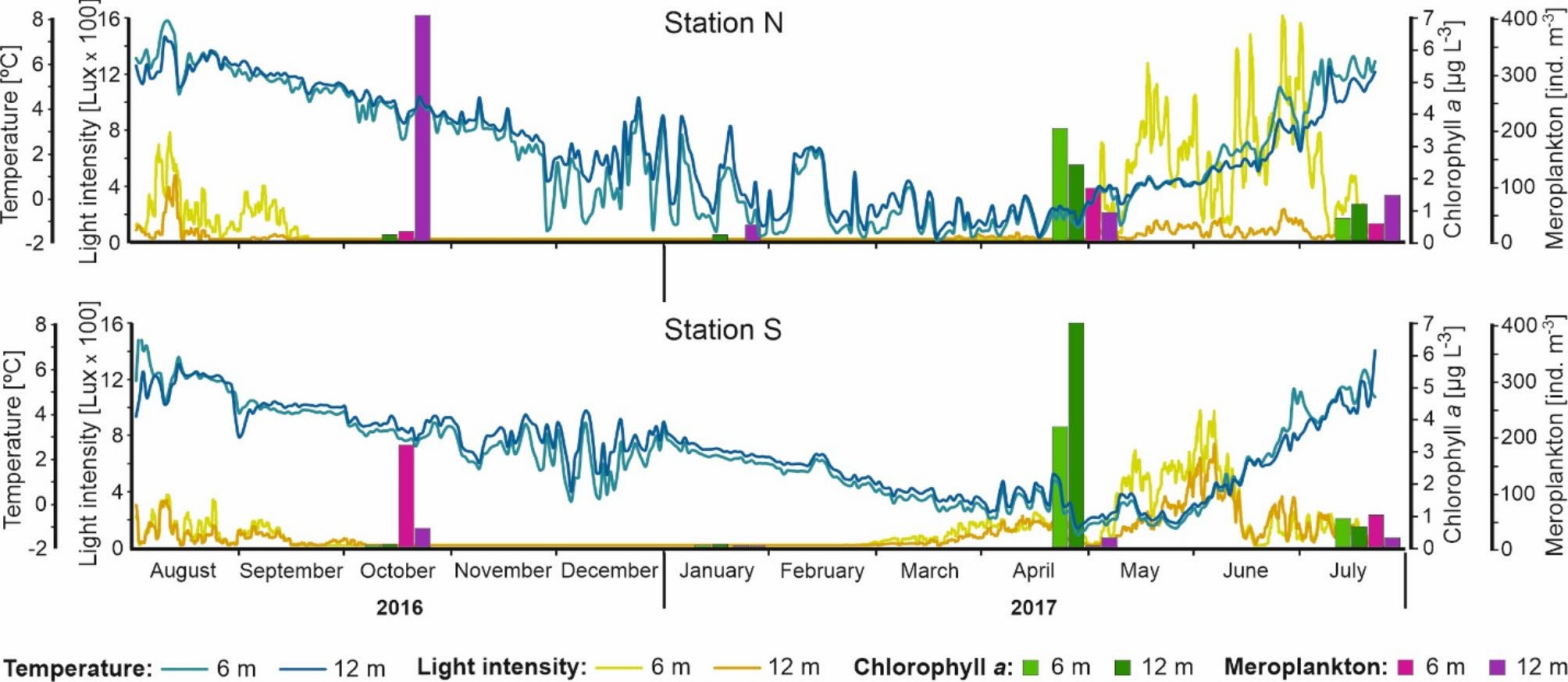
Continuous measurements of temperature and light intensity by underwater loggers attached to the experimental panels from July/August 2016 to July/August 2017, and the concentration of surface chlorophyll *a* and mean meroplankton abundance during the sampling campaigns in Isfjorden at Stations S and N over the depths of 6 and 12 m.

The sea water temperature and light intensity strongly varied seasonally at both stations (Fig. 2). At the beginning of spring, when a gradual increase in sea water temperature and light intensity occurred, the Chl *a* concentration reached its highest value at both stations (3.5 µg Chl *a* L^−1^ at Station N and 6.8 µg Chl *a* L^−1^ at Station S) (Fig. 2). In summer, the sea water temperature was the highest, while the concentration of Chl *a* was much lower (approximately 1 µg Chl *a* L^−1^ at Station N and less than 1 µg Chl *a* L^−1^ at Station S). In the rest of the year, Chl *a* concentration was close to 0 µg Chl *a* L^−1^. The peak mean abundance of potential recruits occurred in the autumn and reached maximum values at Station N at 12 m depth (416 ind. m^−3^) and at Station S at 6 m depth (176 ind. m^−3^). In winter, the abundance of potential recruits was very low (between 0.01 ind. m^−3^ at Station S and 28 ind. m^−3^ at Station N at greater depths). In spring, the abundance of potential recruits was lower at Station S, with a minimum value occurring at 6 m depth (0.86 ind. m^−3^), while at Station N, it reached a maximum value (90 ind. m^−3^) at 6 m depth. Potential recruits reached similar abundances at both stations in summer, but the peaks occurred at different depths; at Station N, the peak was at 12 m depth (83 ind. m^−3^), while at Station S, the peak was at 6 m depth (58 ind. m^−3^).

**Fig. 3:**
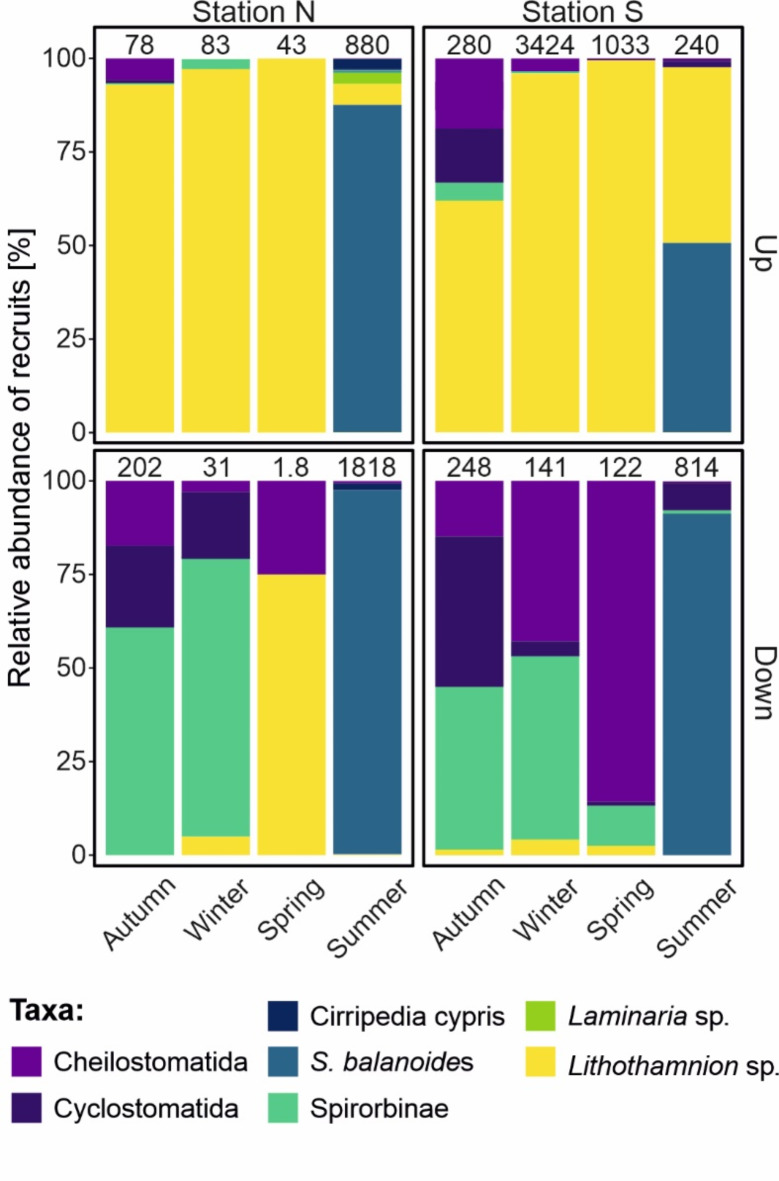
Seasonal variability in the community structure of epibenthic assemblages on experimental plates in Isfjorden (2016–2017) shown for the two sampling stations (N and S) and different plate positions (upward-facing and downward-facing), with the total mean abundance of recruits per plate (pooled depths) given above each bar.

The early-stage epibenthic community structure varied seasonally between stations and between the positions of plates and, to a lesser extent, between sampling depths. The highest number of recruits was observed at Station S on the upward-facing plates in winter (3424 individuals per plate). Over 90% of the recruits were composed of the calcareous alga *Lithothamnion* sp. This taxon dominated on the upward-facing plates in autumn, spring and winter (over 90% of abundance) and on the downward-facing plates in spring (60%) (Fig. 3). In summer, the barnacle *Semibalanus balanoides* reached its highest density, particularly on the downward-facing plates, comprising ca. 90% of the recruits. On the upward-facing plates, the density of barnacles was very high at Station S (80%), while at Station N, they constituted ca. 50% of the assemblage. Juveniles of Spirorbinae polychaetes occurred in the highest numbers on the downward-facing plates in autumn and winter, with a greater percentage at Station N (> 50%). Bryozoans (of two orders, Cheilostomatida and Cyclostomatida) preferably settled on the downward-facing plates, reaching the highest share in autumn, winter and spring at Station S (> 50%).

**Fig. 4:**
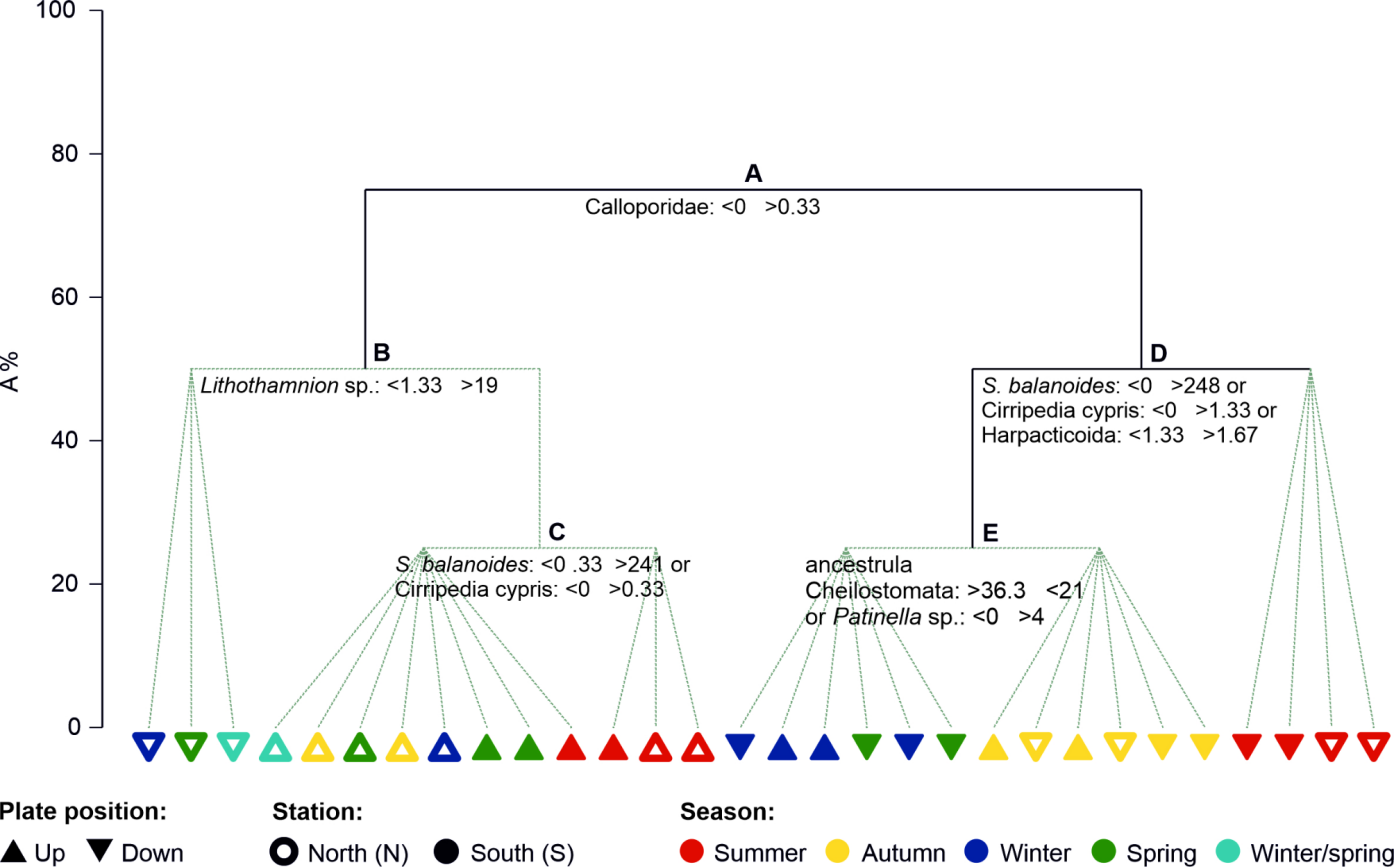
Linkage tree analysis (LINKTREE) showing the partitioning of samples obtained by the five splits (A–E), with corresponding abundance thresholds of the most influential epibenthic taxa. The solid lines represent significant splits according to the SIMPROF tests. The triangles indicate the seasons in which the panels were collected, their positions, and the stations. The winter/spring samples, due to panels not found in winter at 6 m at Station N, were recovered in spring after 6 months.

Differences in the structure of the epibenthic community on the experimental plates were reflected in the partitioning of samples by LINKTREE (Fig. 4). The first, and thus the most important, split A in this divisive clustering was based on the absence or presence of the bryozoan family Calloporidae. The samples without this taxon were then divided according to the number of *Lithothamnion* sp. (split B), which was almost absent in the downward-facing spring and winter samples from Station N and present in the numbers exceeding 19 individuals in the remaining samples. The latter samples were split based on the number of *S. balanoides* or Cirripedia cypris (split C) present almost exclusively on the plates collected in summer at Station N and at 6 m from Station S. However, it is worth noting that splits B and C and the resulting groups of samples were not significant according to the SIMPROF tests. Split D was based on the numbers of *S. balanoides*, Cirripedia cypris or harpacticoids that were present in higher numbers only on the downward-facing plates; these taxa were collected in summer and formed a significant group. The remaining samples from split D were divided by splitting E into two nonsignificant groups: (1) all winter-collected and downward-facing plates from the southern station, which did not contain the bryozoan *Patinella* sp. or had more than 36.3 Cheilostomatida bryozoan ancestrulae; and (2) all autumn-collected samples from Station S, on the downward-facing plates from the N.

In the first series, out of the six RDA models testing six different groups of predictor variables separately, the most powerful was the model based on environmental conditions that explained the highest proportion of total variation in epibenthic taxa (59.84%) (Table 2). In this group of predictors, the highest variation was explained by the position of the experimental plates (21.7%), followed by the light intensity (17.1%), temperature (10.9%), and station and sampling depth. Among the biological variables at the start of panel exposition, those significantly correlated with the epibenthic community were the meroplanktonic larvae Cirripedia nauplii (explaining 27.9% of the variation) and Bryozoa cyphonautes (6%), zooplanktonic *Microsetella* sp. (27.9%), *Pseudocalanus* spp. (5.2%), fish embryos (5.9%) and Cyclopoids (5.1%), as well as the phytoplanktonic *Chaetoceros* spp. (27.9%) and *Gonyaulax gracilis* (7.8%). The biological variables that significantly affected the composition of the epibenthic community during the collection of the experimental panels included Cirripedia cypris (13.9%) and Bryozoa cyphonautes (14.4%), which were considered potential recruits, while the planktonic and demersal animals included *Microsetella* sp. (15.9%), *Limacina helicina* (18.6%), and nematodes (6.4%). Of all the variables tested by forward selection in the subsequent RDAs, the above variables were statistically significant and thus included in the final RDA model used for variation partitioning between abiotic and biotic factors.

**Table 2:** The results of six RDA models, testing different groups of variables separately to explain the variability in epibenthic communities on the experimental plates in Isfjorden, Svalbard 2016–2017, with the list of significant variables, proportion of total variation explained, contribution to explained variation, *pseudo*-F statistics and probability.

Duration/timing	Group of variables	Variable	Explains %	Contribution %	Pseudo-F	*P*
Continuous	Environmental conditions	Position	21.7	35.2	7.8	0.001
		Light intensity	17.1	27.7	7.5	0.001
		Temperature	10.9	17.6	5.6	0.001
		Station	6.3	10.3	3.6	0.001
		Depth	3.9	6.2	2.3	0.029
Start of plates’ exposition	Potential recruits	nauplius Cirripedia	27.9	66.9	10.8	0.001
		cyphonautes Bryozoa	6	14.4	2.5	0.027
	Zooplankton/demersal	*Microsetella* sp.	27.9	44.1	10.8	0.001
		*Pseudocalanus* spp.	5.2	8.3	2.1	0.048
		fish embryo	5.9	9.4	2.5	0.025
		Cyclopoida	5.1	8.1	2.3	0.049
	Phytoplankton	*Chaetoceros* spp.	27.9	44.1	10.8	0.001
		*Gonyaulax gracilis*	7.8	12.3	3.3	0.009
End of plates’ exposition	Potential recruits	cypris Cirripedia	13.9	34.1	4.5	0.001
		Cyphonautes Bryozoa	14.4	35.2	5.4	0.001
	Zooplankton/demersal	*Microsetella* sp.	15.9	25.2	5.3	0.001
		*Limacina helicina*	18.6	29.3	7.6	0.001
		Nematoda	6.4	10.1	2.8	0.028

**Fig. 5:**
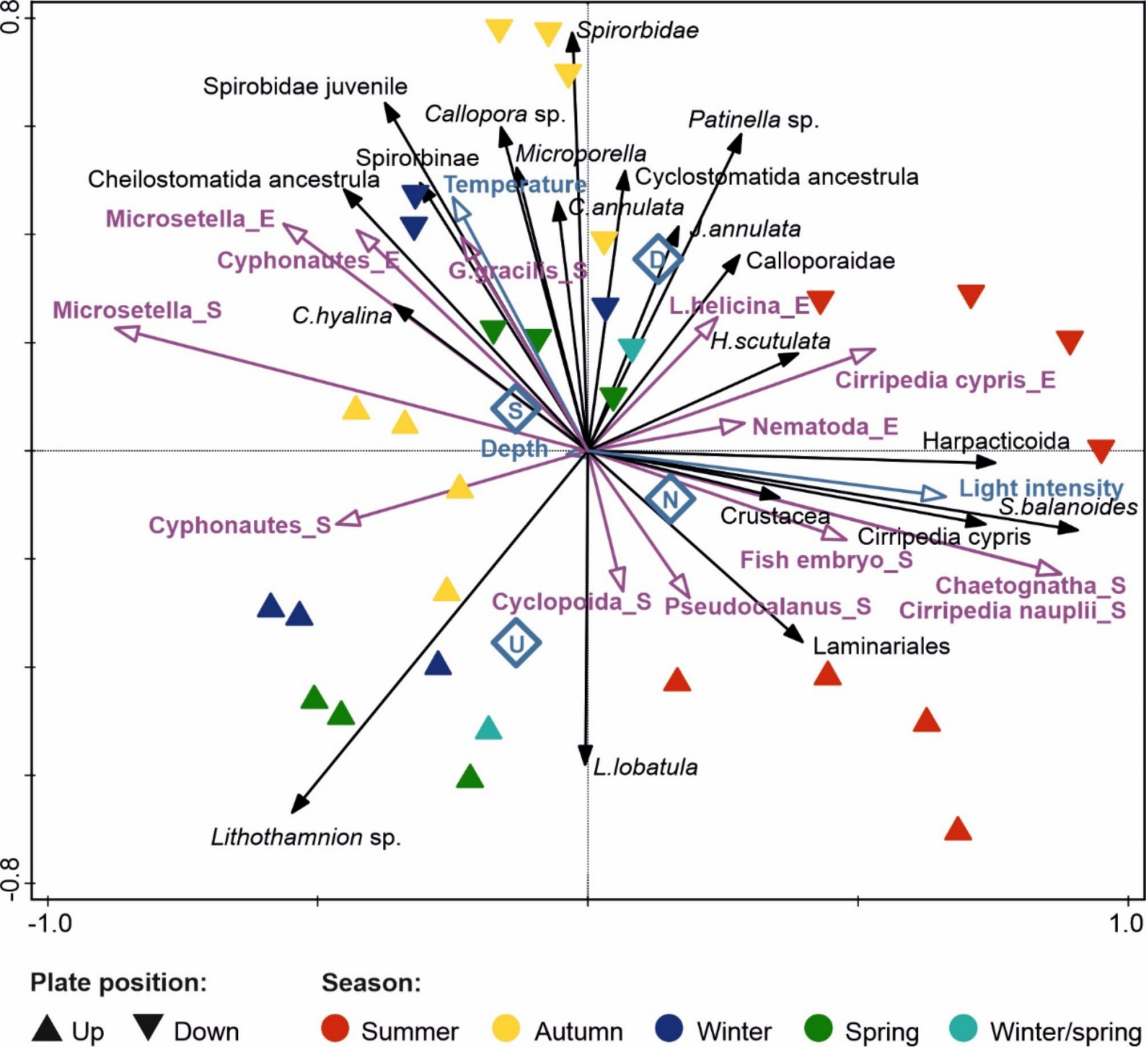
RDA ordination diagram of the 20 best-fitting epibenthic taxa (black arrows) and significant environmental variables: abiotic (blue arrows for the continuous variables; blue diamonds as centroids for the plates positions: downward-facing D and upward-facing U, as well as Stations southern S and northern N), and biotic (purple arrows; “S” are taxa present in the water column at the start of the panels’ exposition, “E” were present at the end). The triangles are centroids for the samples and indicate the seasons in which the panels were collected and their positions.

The ordination plot (Fig. 5), which was derived from the final RDA and was based on predictor variables that significantly influence the epibenthic community (Table 2), indicated that seasonal differences in the recruitment of epibenthic taxa play a major role in the structure of the early epibenthic community. This is manifested by the increasing abundances of crustaceans, including different life stages of Cirripedia and Harpacticoida, gastropods, bivalves, and the brown seaweed *Laminaria* sp., in summer when light intensities were the highest (Fig. 5). Most of the autumn-collected samples were located on the opposite side of the plot. Here, the temperature eigenvector indicated the highest water temperatures, and primarily species and developmental stages of bryozoans were present, such as Cheilostomatida and Cyclostomatida ancestrulas, *Celleporella hyalina*,* Cribilina annulata* and representatives of the Calloporidae family.

The distribution of samples in the ordination plot also showed a clear distinction between taxa preferring different plate positions. Algae such as *Lithothamnion* sp. or *Laminaria* sp., gastropods and the foraminiferan *Lobatula lobatula* preferred UP surfaces, while the majority of the remaining taxa preferred the downward-facing plates. There were also close correlations between meroplanktonic larvae and related taxa, such as Cirripedia nauplii together with *S. balanoides* and Cirripedia cypris at the start of the experimental plate exposition or between the cyphonautes Bryozoa and *C. hyalina* and Cheilostomatida ancestrulae during the collection of the plates.

**Fig. 6:**
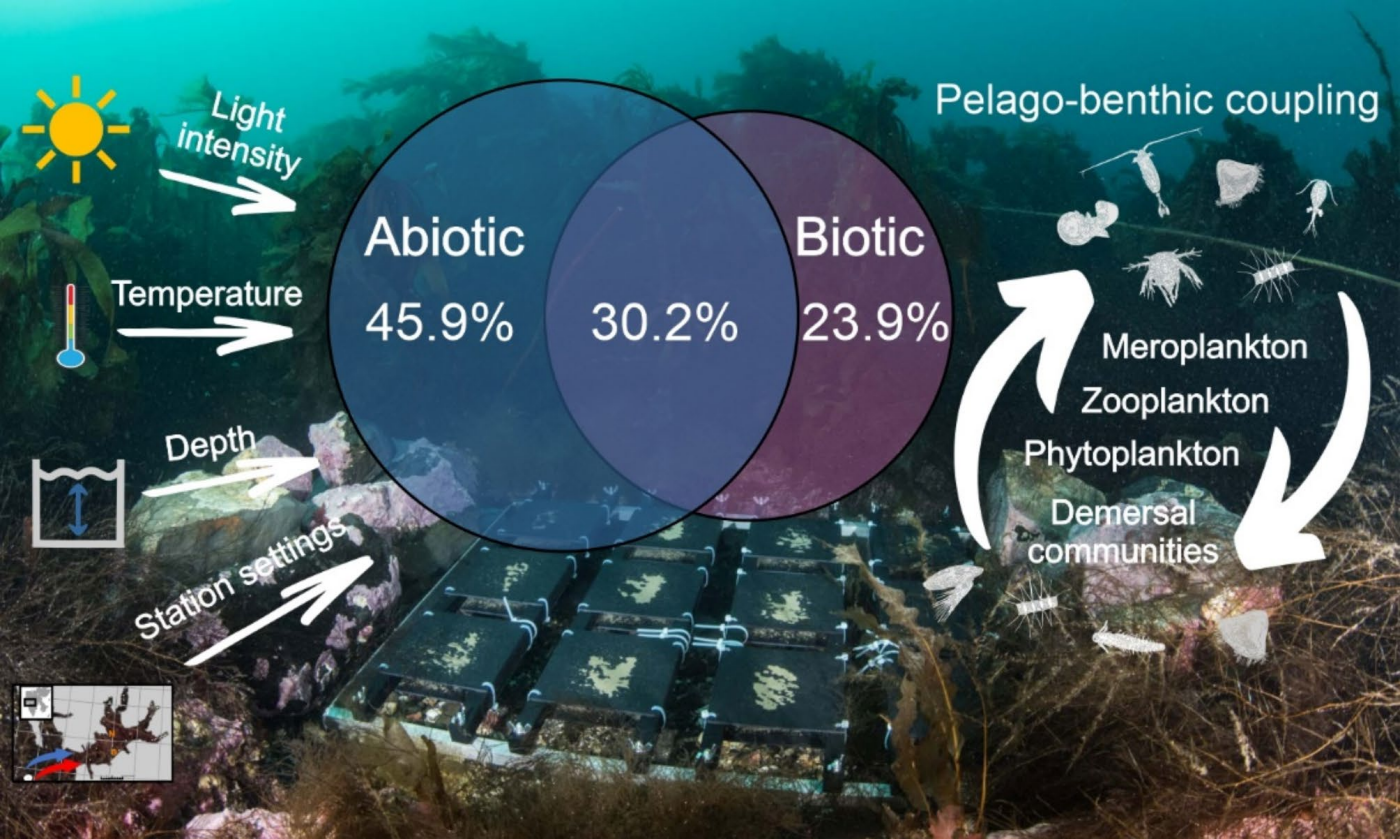
Venn diagram representing the partition of explained variation in the composition and abundance of epibenthic taxa shared between the two groups of significant explanatory variables: (1) abiotic (temperature, light intensity, settlement plate position, depth and station) and (2) biotic (meroplankton and zooplankton at the start of panel exposure and at the end, and phytoplankton at the beginning of panel exposure). Background picture shows the experimental panel’s setting at station S.

The final RDA model with two groups of predictors explained 70.2% of all variation in epibenthic taxa composition, which was partitioned into abiotic variables (45.9% of explained variation) and biotic variables (23.9%), while interactions between these groups of predictors were responsible for 30.2% of the explained variation (Fig. 6). The abiotic variables included temperature, light intensity, settlement plate position, depth and station; among the biotic ones were meroplankton and zooplankton/demersal taxa at the start and in the end of panel exposure, as well as the phytoplankton at the beginning of panel exposure (Table 2).

## Discussion

The recruitment of epibenthic animals and algae in the shallow Arctic coastal zone was driven by a combination of abiotic and biotic factors. The most important environmental drivers were light availability and intensity, which are closely correlated with season and are expressed by the high proportion of variability explained both by the position of the plates and by the light intensity data collected by loggers placed on the experimental panels. Thereafter, sea temperature, site and depth were important factors shaping the settling community. The main split in LINKTREE separated samples according to differences in the epibenthic assemblages on the upper and downward-facing sides of the plates (groups B and D, Fig. 4). The taxon responsible for this grouping was the bryozoan family Calloporidae, which was present on the upper side of plates in very low numbers and was much more common on the bottom side. The position of the plates mimicked a shade effect; the upward-facing plates were exposed to light (especially during midnight sunlight), while the downward-facing plates were sheltered from sunlight. The downward-facing plates also offer a clean surface, not covered by sediments, and a space free from potential competition with algae that need light to grow. Similarly to our experiment, bryozoan larvae have been found to be negatively photoactive, and shaded localities, such as the underside of rocks, were selected for settling^41,42^.

Afterwards, distinct LINKTREE separation between settling communities on the upward-facing plates dominated by the coralline red algae *Lithothamnion* sp. was found in autumn, winter, spring and the barnacles in summer. These findings demonstrate differences in life history strategies and reproductive timing, as the settling of coralline red algae continued to increase during the polar night, which was an exciting new discovery. The common Svalbard coralline alga *Lithothamnion glaciale*^43^ produces reproductive conceptacles containing spores during the winter months in Scotland^44^ and all year round in Greenland^45^. However, before our observations, it was not known whether the spores could colonize new areas during the polar night, the coldest and darkest period of the year. For instance, Adey^46^ noted that for the other subarctic coralline species *Clathromorphum circumscriptum*, a reduced temperature (2–3 °C) and/or reduction in light intensity were critical for asexual reproduction. The higher light intensities and slightly greater sea temperature at Station S than at Station N were primarily due to the exposure of cold, turbid glacial water at the northern location (Fig. 1^23^). The fjord circulation is counterclockwise in Isfjorden, in which surface waters flow along the southern side to the northern side before leaving the fjord and continuing northwards due to the south‒north flowing coastal current and the Coriolis effect^33^. The greater number of algal recruits at Station S (Fig. 3) is most likely the result of variability in environmental conditions connected to the presence of different water masses, of Atlantic and Arctic origin, flowing from the shelf and continuing along the southern coast of Isfjorden (see Fig. 1), with a depth-averaged current near our study site S varying between 0 and 35 cm s^−1^^33^.

The high abundance of settling *S. balanoides* and cyprid larvae in summer (groups C and D in Fig. 4) is directly related to algal food abundance and temperature^47^. Cyprid larvae and young individuals of *S. balanoides* were observed on the settlement plates only between May and July, when the settlement of barnacles occurred, initiating the predominance of young cirripeds (with many cypris larvae attached to the surface) in the summer assemblages on the experimental plates. Our results are in accordance with observations on the timing of metamorphosis from the naupliar to cyprid stage in mid-June in the same fjord^22^, where the duration of nonfeeding cyprid larvae of *Balanus balanus* was estimated to last approximately 1–2 weeks. Although *S. balanoides* is known to occur preferentially in the intertidal zone, this species can also be found submerged into the sublittoral zone (Feyling-Hanssen^48^ and references therein), as in the present study. In the Arctic, *S. balanoides* usually dominates the assemblages on boulder shores, but it can also be present in lower numbers in subtidal areas as observed in other Svalbard fjords, e.g., Hornsund and Kongsfjorden^49^. Interestingly, no *B. balanus* recruits were found in the present study despite Cirripedia larvae, belonging primarily to this species, being found in the same fjord from May-June^22^; remarkably, Barnes and Barnes^50^ also noticed an almost complete absence of *B. balanus* on experimental panels, while other cirriped species (*S. balanoides* and *B. crenatus*) were among common settlers in the Clyde Sea Area (Scotland).

The last LINKTREE clusters separated winter samples and downward-facing spring samples at Station S from all samples collected in autumn based on the greater occurrence of the bryozoan Cheilostomatida ancestrulae and lack of the bryozoan *Patinella* sp. (order Cyclostomatida) in the first group. There was a shift in the timing of settlement of ancestrulae belonging to the orders Cheilostomatida and Cyclostomatida indicating that species of particular bryozoan orders have a preferred season for settlement: the first occurred more commonly in winter and spring, while the latter in autumn and summer. The majority of bryozoans possess short-lived, demersal, lecithotrophic larvae that metamorphose within a few hours of liberation^51^. Consequently, these species are extremely difficult to collect in the field, and all attempts to collect them have failed^23,27^. During the present study, neither were they present in the water column nor in the near-sea bed layer sampled with the use of a hand-held suction pump operated around the bottom crevices by a SCUBA diver^23^. The most likely reasons are the very short period of time when the larvae creep/glide on the surface while searching for suitable attachment substrata and their preference for shaded places such as those beneath boulders, which further hinder sampling. On the other hand, pelagic, longer-lived cyphonautes larvae were present in the autumn meroplankton samples. Some correlations between cyphonautes Bryozoa and the cheilostome *Celleporella hyalina* or Cheilostomatida ancestrulae, as indicated by RDA (Fig. 5), could indicate the taxonomic affiliation of meroplanktonic larvae, which had been identified only at higher taxonomic levels due to their difficult identification at the larval stage. However, *C. hyalina* broods are known to be lecithotrophic larvae^52^. Therefore, the cyphonautes may belong to an unidentified cheilostome ancestrulae; thus, we cannot connect pelagic larvae with bryozoan recruits on panels. DNA barcoding could shed more light on the larval species pool and connectivity between the pelagic and benthic phases.

According to the variation partitioning, physical drivers such as the position of panels, light intensity, temperature, location of the sampling stations and depth were responsible for 45.9% of the variability in recruiting biota. Biotic conditions also played an important role (such as the presence of meroplanktonic larvae, thus potential recruits, and phyto- and zooplanktonic taxa, which may have acted as potential food sources), which explained 23.9% of the variability. Good examples of the connection between meroplankton and recruits on the experimental panels were the early larval stages of Cirripedia (nauplii), which were present in the water column at the start of the panels exposition, and their older larval stage, cyprids, at the end of exposition; these occurrences were correlated with the recruitment of *S. balanoides* and the presence of cyprids on the plates in summer. This reflects the biological development cycle that is strictly triggered by spring phytoplankton blooms and increased temperatures^4,22^.

However, the relationships between holoplankton/demersal or phytoplankton taxa, which were treated as potential food sources, and the recruitment of epibenthos are unclear. Among the most influential phytoplanktonic taxa were the diatom *Chaetoceros* spp. and the dinoflagellate *Gonyaulax gracilis*. Furthermore, the zooplanktonic copepods with significant impacts were the harpacticoid *Microsetella* sp., the calanoid *Pseudocalanus* spp. and species belonging to the order Cyclopoida. Phytoplankton provide the best food source for suspension feeders, including bryozoans and barnacles^53^. In particular, small flagellates (less than 5 μm in diameter) and diatoms are the preferred items for a variety of bryozoan species. *Chaetoceros* spp. are also known to serve as a food source for calanoid copepods^54^ and are considered to be among the best foods for the larvae of commercially cultivated bivalves^55^. The ecological role of *Gonyaulax gracilis* is poorly understood, and additional evidence is needed to document its connection with epibenthic development. The pelagic harpacticoid *Microsetella norvegica* plays an important role in food webs as a grazer of particulate-related food sources^56^. *Pseudocalanus* spp. and *Limacina helicina* may trap smaller copepods, juveniles of conspecifics, diatoms and flagellates^57,58^. In our study, it seems that there was no direct relationship between the occurrence of these taxa and epibenthic recruitment. However, when analysing biotic variables, it should be taken into account that environmental conditions influence not only the epibenthic community but also organisms living in the water column or above the seabed, as indicated by the high proportion of variation shared between abiotic and biotic variables according to the variation partitioning analysis.

Predation and competition for space and/or food are other possible biotic drivers that can have a negative impact on developing epibenthic assemblages by causing early post-settlement mortality in recruits^59^. We did not observe any direct evidence of predation or grazing on recruiting assemblages or competition for space, most likely related to the relatively short settling time (3 months). Nevertheless, some tracks of chitons and echinoids were observed on the panels (personal observations), and together with the inter- and intraspecific interactions between recruiting individuals, these tracks will be analysed further.

The structure of benthic communities is determined by the supply of recruits, transport mechanisms, settlement success and postlarval processes, in which mortality in both the larval and settled stages is very important. According to Thorson’s assumptions^60^, pelagic larvae undergo extremely high mortality, with only 0.1% of pelagic larvae achieving settlement and 1% of metamorphosed juveniles reaching sexual maturity. These phenomena may in part be responsible for differences between the composition of planktonic communities present in the water column and the structure and composition of epibenthic animals and algae in the shallow Arctic coastal zone. Isfjorden is also a highly advective system, meaning that recruitment is not only determined by local reproduction but also supplied by distant populations, especially in the case of species that have long-lived pelagic larval stages, such as barnacles.

The rate of climate-induced changes in the Arctic and resulting continuous transport of organisms of boreal origin northwards, combined with higher sea temperatures and less sea ice allowed the expansion of sub-Arctic and boreal populations in the Arctic^10,11^. This may lead to a spatial decoupling between larval production and local settlement^15^, therefore we believe that there is a need of studies, similar to ours and preferably lasting longer, that would contribute to a better understanding of complex interactions between the changing environment and biotic components of the Arctic marine ecosystems, particularly the recruitment of sessile benthic biota.

## Summary and conclusions

1. The present study adds new knowledge about pelagic-benthic coupling and factors driving dispersal and epibenthos recruitment in shallow hard-bottom Arctic communities, which are still poorly known, but important taking into account that most benthic organisms possess a planktonic propagule (larva or spore), which enables them to spread over large distances before settlement (Fig. 6).
2. Settlement of sessile benthic biota highly depends on light intensity and water temperature; these physical drivers, together with the position of the plates, location of the sampling stations and depth, were responsible for 46% of variability in recruiting epibenthic biota. Biotic factors such as the presence of meroplanktonic larvae and phyto- and zooplanktonic taxa explained 24% of the variability.
3. Coralline red algae dominated on the upward-facing plates, while polychaetes and bryozoans on the downward-facing plates, with the exception of summer, when *S. balanoides* outcompeted the other recruits on both sides of the plates. High seasonal variation in the recruiting organisms was particularly common on the downward-facing plates. The autumn and winter plates were colonized mainly by Spirorbinae and bryozoans; the spring plates were colonized by *Lithothamnion* sp. and bryozoans at the northern station and bryozoans and Spirorbinae at the southern station. In summer, the plates were mainly overgrown by *S. balanoides* juveniles, regardless of the site.
4. The high numbers of crustose coralline algae *Lithothamnion* sp. found on plates during the polar night indicated that solar radiation is not required to initiate algal spore release and settlement; to our knowledge, this is the first observation that, at high latitudes, coralline algae settle on available substrates during the polar night.
5. The connectivity between pelagic meroplanktonic larvae and settling benthic biota was best exhibited by the seasonal sequence of cirriped life stages, followed by that of cyphonautes larvae, which may be unidentified Cheilostomatida present on the panels.

## Data Availability

The datasets generated during and/or analysed during the current study are available from the corresponding author on reasonable request.

## References

[1] Pineda-Metz, S. E. A. Benthos-pelagos interconnectivity: Antarctic shelf examples. In *YOUMARES 9—The Oceans: Our Research, Our Future* (eds Jungblut, S. et al.) 211–223 (Springer, 2020).

[2] Fetzer, I. & Arntz, W. E. Reproductive strategies of benthic invertebrates in the Kara Sea (Russian Arctic): Adaptation of reproduction modes to cold water. *Mar. Ecol. Prog. Ser.* **356**, 189–202. 10.3354/meps07271 (2008).

[3] Grosberg, R. K. & Quinn, J. F. The genetic control and consequences of kin recognition by the larvae of a colonial marine invertebrate. *Nature* **322**, 456–459 (1986).

[4] Highfield, J. M. et al. Seasonal dynamics of meroplankton assemblages at station L4. *J. Plankton Res.* **32**, 681–691 (2010).

[5] Daase, M., Berge, J., Søreide, J. E. & Falk-Petersen, S. Ecology of Arctic pelagic communities. In *Arctic Ecology*, 219–259 (2021).

[6] Shanks, A. L. Pelagic larval duration and dispersal distance revisited. *Biol. Bull.* **216**, 373–385 (2009). 19556601 10.1086/BBLv216n3p373

[7] Meadows, P. S. & Campbell, J. I. Habitat selection by aquatic invertebrates. *Adv. Mar. Biol.* **10**, 271–382 (1972).

[8] Clough, L. M. et al. Meroplankton abundance in the Northeast Water Polynya: Insights from oceanographic parameters and benthic abundance patterns. *J. Mar. Syst.* **10**, 343–357 (1997).

[9] Polyakov, I. V. et al. Borealization of the Arctic Ocean in response to anomalous advection from sub-Arctic seas. *Front. Mar. Sci.* **7**, 491 (2020).

[10] Wassmann, P., Duarte, C. M., Agusti, S. & Sejr, M. K. Footprints of climate change in the Arctic marine ecosystem. *Glob. Change Biol.* **17**, 1235–1249 (2011).

[11] Weydmann, A. et al. Shift towards the dominance of boreal species in the Arctic: Inter-annual and spatial zooplankton variability in the West Spitsbergen Current. *Mar. Ecol. Prog. Ser.* **501**, 41–52. 10.3354/meps10694 (2014).

[12] Walczowski, W. & Piechura, J. New evidence of warming propagating toward the Arctic Ocean. *Geophys. Res. Lett.* **33**, L12601 (2006).

[13] Stempniewicz, L. et al. Advection of Atlantic water masses influences seabird community foraging in a high-Arctic fjord. *Prog. Oceanogr.* **193**, 102549. 10.1016/j.pocean.2021.102549 (2021).

[14] Strzelewicz, A., Przyborska, A. & Walczowski, W. Increased presence of Atlantic Water on the shelf south-west of Spitsbergen with implications for the Arctic fjord Hornsund. *Prog. Oceanogr.* **200**, 102714 (2022).

[15] Ershova, E. A. et al. Diversity and distribution of meroplanktonic larvae in the Pacific Arctic and connectivity with adult benthic invertebrate communities. *Front. Mar. Sci.* **6**, 490. 10.3389/fmars.2019.00490 (2019).

[16] Reitzel, A. M., Miner, B. G. & McEdward, L. R. Relationships between spawning date and larval development time for benthic marine invertebrates: a modeling approach. *Mar. Ecol. Prog. Ser.* **280**, 13–23 (2004).

[17] Byrne, M. & Przeslawski, R. Multistressor impacts of warming and acidification of the ocean on marine invertebrates’ life histories. *Integr. Comp. Biol.* **53**, 582–596 (2013). 23697893 10.1093/icb/ict049

[18] Atkinson, D. Temperature and organism size-a biological law for ectotherms?. *Adv. Ecol. Res.* **25**, 1–58 (1994).

[19] Sheridan, J. A. & Bickford, D. Shrinking body size as an ecological response to climate change. *Nat. Clim. Change* **1**, 401–406 (2011).

[20] Stübner, E. I., Søreide, J. E., Reigstad, M., Marquardt, M. & Blachowiak-Samolyk, K. Year-round meroplankton dynamics in high-Arctic Svalbard. *J. Plankton Res.* **38**, 522–536. 10.1093/planktfbv124 (2016).

[21] Brandner, M. M., Stübner, E., Reed, A. J., Gabrielsen, T. M. & Thatje, S. Seasonality of bivalve larvae within a high Arctic fjord. *Polar Biol.* **40**, 263–276 (2017).

[22] Walczyńska, K. S., Søreide, J. E., Weydmann-Zwolicka, A., Ronowicz, M. & Gabrielsen, T. M. DNA barcoding of Cirripedia larvae reveals new knowledge on their biology in Arctic coastal ecosystems. *Hydrobiologia* **837**, 149–159. 10.1007/s10750-019-3967-y (2019).

[23] Weydmann-Zwolicka, A. et al. Meroplankton seasonal dynamics in the high Arctic fjord: Comparison of different sampling methods. *Prog. Oceanogr.* **190**, 102484. 10.1016/j.pocean.2020.102484 (2021).

[24] Barnes, D. K. A. & Kuklinski, P. Low colonisation on artificial substrata in arctic Spitsbergen. *Polar Biol.* **29**, 65–69 (2005).

[25] Meyer, K. S. et al. Recruitment of benthic invertebrates in high Arctic fjords: Relation to temperature, depth, and season. *Limnol. Oceanogr.* **62**, 2732–2744. 10.1002/lno.10602 (2017).

[26] Sowa, A. et al. Factors shaping epibionts recruitment in the high Arctic (Isfjorden, Spitsbergen): A year-round investigation using experimental plates. *Estuar. Coast. Shelf Sci.* **283**, 108281. 10.1016/j.ecss.2023.108281 (2023).

[27] Kuklinski, P. et al. Seasonality of occurrence and recruitment of Arctic marine benthic invertebrate larvae in relation to environmental variables. *Polar Biol.* **36**, 549–560. 10.1007/s00300-012-1283-3 (2013).

[28] Meyer-Kaiser, K. S. et al. Larval dispersal and recruitment of benthic invertebrates in the Arctic Ocean. *Prog. Oceanogr.* **203**, 102776 (2022).

[29] Nilsen, F., Cottier, F., Skogseth, R. & Mattsson, S. Fjord–shelf exchanges controlled by ice and brine production: the interannual variation of Atlantic Water in Isfjorden, Svalbard. *Cont. Shelf Res.* **28**, 1838–1853 (2008).

[30] Pawłowska, J., Włodarska-Kowalczuk, M., Zajączkowski, M., Nygård, H. & Berge, J. Seasonal variability of meio-and macrobenthic standing stocks and diversity in an Arctic fjord (Adventfjorden, Spitsbergen). *Polar Biol.* **34**, 833–845 (2011).

[31] Muckenhuber, S., Nilsen, F., Korosov, A. & Sandven, S. Sea ice cover in Isfjorden and Hornsund, Svalbard (2000–2014) from remote sensing data. *Cryosphere* **10**, 149–158 (2016).

[32] Svendsen, P. The algal vegetation of Spitsbergen. A survey of the marine algal flora of the outer part of Isfjorden. *Norsk Polarinst.* **116**, 1–58 (1959).

[33] Skogseth, R. et al. Variability and decadal trends in the Isfjorden (Svalbard) ocean climate and circulation—An indicator for climate change in the European Arctic. *Prog. Oceanogr.* **187**, 102394 (2020).

[34] Kuklinski, P. et al. Experimental apparatus for investigating colonization, succession and related processes of rocky bottom epifauna. *Cont. Shelf Res.* **233**, 104641 (2022).

[35] Stanwell-Smith, D., Peck, L. S., Clarke, A., Murray, A. W. & Todd, C. D. The distribution, abundance and seasonality of pelagic marine invertebrate larvae in the maritime Antarctic. *Philos. Trans. R. Soc. Lond. B* **354**, 471–484 (1999).

[36] Utermöhl, H. Zur vervollkommnung der quantitativen phytoplankton-methodik: Mit 1 Tabelle und 15 abbildungen im Text und auf 1 Tafel. *Int. Ver. Theor. Angew. Limnol.* **9**, 1–38 (1958).

[37] Jeffrey, S. W. & Humphrey, G. F. New spectrophotometric equations for determining chlorophyll a, b, c1 and c2 in higher plants, algae, and natural phytoplankton. *Biochem. Physiol. Pflanzen* **167**, 191–194 (1975).

[38] Postel, L., Fock, H. & Hagen, W. Biomass and abundance. In *ICES Zooplankton Methodology Manual* (eds Harris, R. P. et al.) 83–192 (Academic Press, 2000).

[39] Clarke, K. R., Somerfield, P. J. & Gorley, R. N. Testing of null hypotheses in exploratory community analyses: Similarity profiles and biota-environment linkage. *J. Exp. Mar. Biol. Ecol.* **366**, 56–69. 10.1016/j.jembe.2008.07.009 (2008).

[40] Ter Braak, C. J. F. & Šmilauer, P. *Canoco Reference Manual and User’s Guide: Software for Ordination, Version 5.0* (Microcomputer Power, Ithaca, 2012).

[41] Ryland, J. S. Experiments on the influence of the light on the behaviour of polyzoan larvae. *J. Exp. Biol.* **37**, 783–800 (1960).

[42] Kuklinski, P., Balazy, P., Nowak, M. & Bielecka, L. Factors controlling initial development of Polar bryozoan assemblages. *Studi Trent. Sci. Nat.* **94**, 145–151 (2014).

[43] Teichert, S. et al. Arctic rhodolith beds and their environmental controls (Spitsbergen, Norway). *Facies* **60**, 15–37 (2014).

[44] Hall-Spencer, J. M. Biological studies on nongeniculate Corallinaceae. PhD thesis, University of London (1994).

[45] Rosenvinge, L. K. The marine algae of Denmark. Part II. Rhodophyceae II. (Cryptonemiales. *Kongel. Danske Vidensk. Selsk. Skr. 7 Raekke Nat. Mat. Afd* **7**, 155–283 (1917).

[46] Adey, W. H. Temperature control of reproduction and productivity in a subarctic coralline alga. *Phycologia* **12**, 111–118 (1973).

[47] Barnes, H. & Barnes, M. The rate of development of the embryos of *Balanus balanoides* (L.) from a number of European and American populations and the designation of local races. *J. Exp. Mar. Biol. Ecol.* **24**, 251–269 (1976).

[48] Feyling-Hanssen, R. W. The barnacle *Balanus balanoides* (Linne, 1766) in Spitsbergen. *Norsk Polarinst.* **98**, 1–64 (1953).

[49] Kuklinski, P. & Barnes, D. K. A. Structure of intertidal and subtidal assemblages in Arctic vs temperate boulder shores. *Pol. Polar Res.* **29**, 203–218 (2008).

[50] Barnes, H. & Barnes, M. The general biology of *Balanus balanus* (L.) Da Costa. *Oikos* **5**, 63–76 (1954).

[51] Ryland, J. S. The identity of some cyphonautes larvae (Polyzoa). *J. Mar. Biol. Assoc. U. K.* **44**, 645–654 (1964).

[52] Cancino, J. M., Hughes, R. N. & Ramirez, C. Environmental cues and the phasing of larval release in the bryozoan *Celleporella hyalina* (L.). *Proc. R. Soc. Lond. B* **246**, 39–45 (1991).

[53] Winston, J. E., Woollacott, R. M. & Zimmer, R. L. Feeding in marine Bryozoans. *Biol. Bryozoans* **233**, 271 (1977).

[54] Bradstreet, M. W. & Cross, W. E. Trophic relationship at high Arctic ice edges. *Arctic* **35**, 1–12 (1982).

[55] Enright, C. T., Newkirk, G. F., Craigie, J. S. & Castell, J. D. Evaluation of phytoplankton as diets for juvenile *Ostrea edulis* L. *J. Exp. Mar. Biol. Ecol.* **96**, 1–13 (1996).

[56] Koski, M., Kiørboe, T. & Takahashi, K. Benthic life in the pelagic: Aggregate encounter and degradation rates by pelagic harpacticoid copepods. *Limnol. Oceanogr.* **50**, 1254–1263 (2005).

[57] Gilmer, R. W. & Harbison, G. R. Diet of *Limacina helicina* (Gastropoda: Thecosomata) in Arctic waters in midsummer. *Mar. Ecol. Prog. Ser.* **77**, 125–134 (1991).

[58] Turner, J. T. The feeding ecology of some zooplankters that are important prey items of larval fish. *NOAA Technical Report NMFS* **7** (1984).

[59] Hunt, H. L. & Scheibling, R. E. Role of early post-settlement mortality in recruitment of benthic marine invertebrates. *Mar. Ecol. Prog. Ser.* **155**, 269–301. 10.3354/meps155269 (1997).

[60] Thorson, G. Some factors influencing the recruitment and establishment of marine benthic communities. *Neth. J. Sea Res.* **3**, 267–293 (1966).

